# Experimental vibration data collected for a belt drive system under different operating conditions

**DOI:** 10.1016/j.dib.2023.109175

**Published:** 2023-04-27

**Authors:** Ramy M． Khalifa, Soumaya Yacout, Samuel Bassetto, Yasser Shaban

**Affiliations:** a Department of Mathematics and Industrial Engineering, École Polytechnique de Montréal, Montréal, Québec H3T 1J4 Canada; b Department of Mechanical Design Engineering, Helwan University, Cairo, Egypt

**Keywords:** Vibration signal, Belt drive system, Condition monitoring, Belt conditions

## Abstract

Vibration analysis is the cornerstone of vibration-based condition monitoring that analyzes a vibration signal, detects faults or anomalies, and diagnoses the operating conditions of a belt drive system. This data article contains experiments that collect vibration signals of a belt drive system at different levels of speed and pretension of the belt under varying operating conditions. The collected dataset includes low, medium, and high operating speeds at three levels of the belt’s pretensioned values. This article covers three operating conditions: normal or healthy operation using a healthy belt, unbalanced operation by adding unbalanced weight to the system, and abnormal operation using a faulty belt. The collected data provides an understanding of the performance of the belt drive system during its operation to identify the root cause of an anomaly when detected.

**Specifications Table**

**Table:** 

Subject	Mechanical Engineering, Industrial Engineering
Specific subject area	Vibration-based condition monitoring and vibration analysis of a belt drive system under different operating conditions
Type of data	Tables in.txt files and figures in.JPG files
How data were acquired	The vibration data was collected by data acquisition system (Accelerometers, Amplifier, and USB data acquisition card) during system operation. The operating speed of the system are controlled and maintained by a speed controller. The pretension value of the belt is adjusted by a using pretension gauge.
Data format	Raw
Parameters for data collection	The data was acquired based on three experimental settings: healthy operation of the system, presence of unbalanced weight, and faulty operation. All of these settings have three parameters in which the experiments are carried out at different operating speeds and pretension values of the belt, in addition to the value of adding weights in case the unbalanced weight settles.
Description of data collection	The data is of vibration signals that were collected by using data acquisition system through the two accelerometers mounted on the test rig. Then, the data was transmitted to a laptop for analysis.
Data source location	Data was obtained from the test rig shown in Fig. 1 in: Institution: Faculty of Engineering - Department of Mechanical Design - Helwan university
	City: Cairo
	Country: Egypt
Data accessibility	The data is available in the Mendeley repository at: https://data.mendeley.com/datasets/jf8v2ndydr/1

## Value of the Data

• The data represents the vibration signals collected from the belt drive system under healthy and faulty conditions. This data is for a commonly used system in various industrial applications.
• The data is useful to the researcher and practitioners in mechanical and industrial engineering, to analyze the vibration signals of the belt drive system and to determine its characteristics under healthy and faulty conditions.
• The data can be used for online condition process monitoring in order to detect and diagnose any anomaly or faulty condition in the system. It can be used to evaluate developed machine learning approaches that distinguish the conditions of the belt drive system.

## 1 Data Description

The belt drive system is widely used in different industrial applications for power transmission such as conveyors, machine tools, and motors [1]. It consists of a motor, shaft(s), bearings, belt(s), and driver and driven pulleys [2]. The system operates at different speeds and transmits power using a pretension of the belt. The system is used to produce different types of anomalies that are developed due to several abnormal sources of vibrations in the system, such as cut or damage of the belt, unbalance problems, and misalignment [3], [4], [5].

This article comprises the experiments that collect vibration signals of the belt drive system at different levels of speed and pretension of the belt under different conditions of healthy belt, faulty belt, and the presence of unbalanced weight. The vibration signals are collected from accelerometers attached to the driver and driven pulleys. The collected data includes 17 levels of speed; 400 to 2000 RPM by step of 100; three levels of pretension values, 70, 110, and 150 N, and two levels, according to whether there was the presence or absence of unbalanced weight. The data is from three different operating conditions: normal operation using a healthy belt, unbalanced operation by adding weights that cause imbalance in the system, and anomalous operation from using a faulty belt. Each experiment has been repeated three times, which resulted in 459 runs.

This article is accompanied by nine folders and its name is given as “T-BC-W” where T, BC, W denote the belt pretension in newton, identification of the belt condition, and absence (W=0) or presence (W=U) of unbalanced weight, respectively. Thus, the data contains the following folders:

• Data 70-H-0: The vibration signals are collected from the belt drive system when the belt is pre-tensioned by 70 N and in healthy condition in addition to the absence of unbalanced weight at all levels of speeds.
• Data 110-H-0: The vibration signals are collected from the belt drive system when the belt is pre-tensioned by 110 N and in healthy condition in addition to the absence of unbalanced weight at all levels of speed.
• Data 150-H-0: The vibration signals are collected from the belt drive system when the belt is pre-tensioned by 150 N and in healthy condition in addition to the absence of unbalanced weight at all levels of speed.
• Data 70-F-0: The vibration signals are collected from the belt drive system when the belt is pre-tensioned by 70 N and in the faulty condition in addition to the absence of unbalanced weight at all levels of speed.
• Data 110-F-0: The vibration signals are collected from the belt drive system when the belt is pre-tensioned by 110 N and in the faulty condition in addition to the absence of unbalanced weight.
• Data 150-F-0: The vibration signals are collected from the belt drive system when the belt is pre-tensioned by 150 N and in the faulty condition in addition to the absence of unbalanced weight at all levels of speed.
• Data 70-H-U: The vibration signals are collected from the belt drive system when the belt is pre-tensioned by 70 N and in healthy condition in addition to the presence of unbalanced weight at all levels of speed.
• Data 110-H-U: The vibration signals are collected from the belt drive system when the belt is pre-tensioned by 110 N and in healthy condition in addition to the presence of unbalanced weight at all levels of speed.
• Data 150-H-U: The vibration signals are collected from the belt drive system when the belt is pre-tensioned by 150 N and in healthy condition in addition to the presence of unbalanced weight at all levels of speed.

Each folder contains 51 TXT files and 51 figures in JPG format that describe each operating condition at different speeds. Both TXT and JPG have the same names. Each TXT file contains 10,000 samples. Since each experiment run has been repeated three times, every three files represent the vibration signals that are collected from the operation of the system at the same speed as in Table 1.

**Table 1:** Files and figures description.

TXT/JPG files	Speed, RPM
1 to 3	400
4 to 6	500
7 to 9	600
10 to 12	700
13 to 15	800
16 to 18	900
19 to 21	1000
22 to 24	1100
25 to 27	1200
28 to 30	1300
31 to 33	1400
34 to 36	1500
37 to 39	1600
40 to 42	1700
43 to 45	1800
46 to 48	1900
49 to 51	2000

## 2 Experimental Design, Materials and Methods

**Fig. 1:**
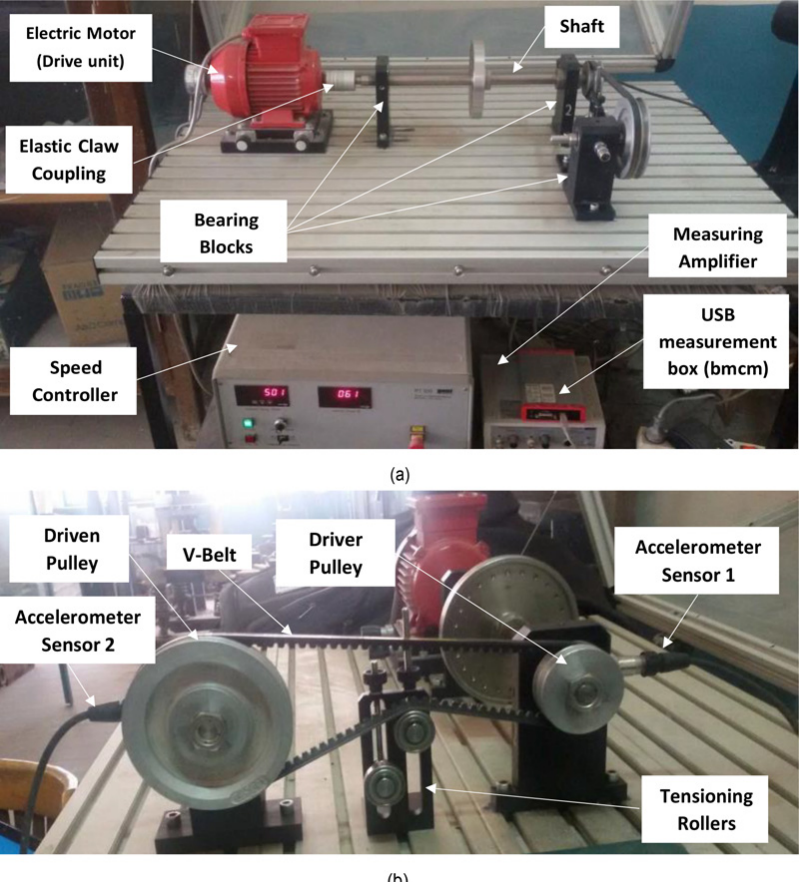
G.U.N.T machinery diagnostic system (PT 500.14) Description.

**Fig. 2:**
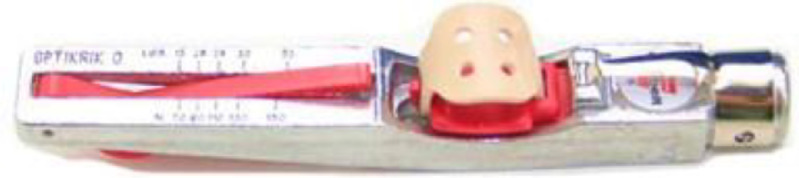
The pretension gauge.

The experiments are performed using the belt drive kit of the G.U.N.T machinery diagnostic system (PT 500.14) [6] as depicted in Fig. 1. The key component of the experiment is a base, unit G.U.N.T (PT 500), [7] that consists of an electric motor as the rotating equipment, a shaft, and two bearing blocks. The speed of the electric motor “*N*” is adjusted by a speed controller. An elastic coupling is considered as the connection between the motor and the shaft where it is used to avoid misalignment and increase the flexibility of the shaft. The two bearing blocks have a ball bearing type that supports the shaft. On the other hand, the G.U.N.T (PT 500.14) consists of a per-tensioned V-belt that connects small driver and large driven pulleys. The diameter of the small driver pulley is 63 mm which is connected to the shaft of the G.U.N.T (PT 500). The V-belt is SPZ type with a length of 912 mm and width of 10 mm. It is the machine element that transmits power to the large driven pulley, which has a diameter of 125 mm. The pretension of the belt “*T*” is adjusted using tensioning rollers. A pretension gauge is used to measure the value of *T* as shown in Fig. 2.

**Fig. 3:**
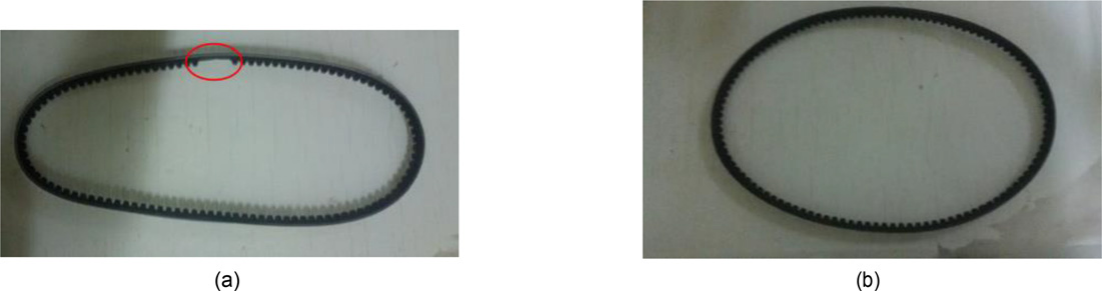
G.U.N.T (PT 500.14) - the healthy and faulty belts.

**Fig. 4:**
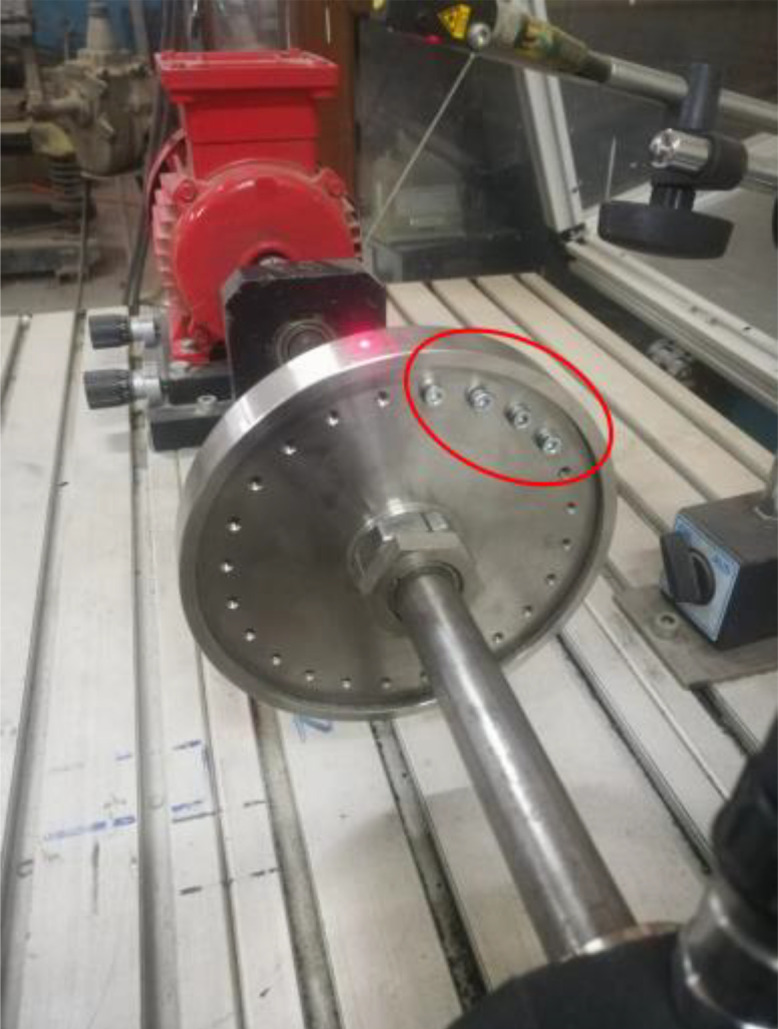
G.U.N.T (PT 500.14) - presence of unbalanced weights.

The Vibration signals are collected using a Data Acquisition system. This has two piezoelectric accelerometers (IMI 603C01); accelerometers 1 and 2 are attached to the bearing block of the driver and driven pulley, respectively, in a horizontal direction using studs. They are used to measure the signals during the experimental run. An amplifier is used to amplify these signals. The output signals from the amplifier are digitalized using a USB data acquisition card (bmcm) and the collected signals are transferred to the *LabVIEW* script installed on a laptop for further analysis. The experiments are conducted to investigate the characteristics of the vibration signals for the belt drive system under three levels of *T* (70, 110, and 150 N), absence and presence of unbalanced weight, using healthy and faulty belts at 17 levels of speed *N* that ranges from 400 to 2000 RPM in step 100. Figs. 3 and 4 show the presence of unbalanced weight, and the healthy and faulty belts.

## Ethics Statement

The author duly adhered to the ElSEVIER “Ethics in Publishing” policy.

## CRediT authorship contribution statement

**Ramy M． Khalifa:** Conceptualization, Data curation, Methodology, Writing – original draft, Writing – review & editing. **Soumaya Yacout:** Investigation, Supervision, Visualization, Writing – original draft, Writing – review & editing. **Samuel Bassetto:** Supervision, Writing – review & editing. **Yasser Shaban:** Visualization, Writing – review & editing.

## Declaration of Competing Interest

The authors declare that they have no known competing financial interests or personal relationships which have, or could be perceived to have, influenced the work reported in this article.

## Data Availability

Experimental vibration data collected for a belt drive system under different operating conditions (Original data) (Mendeley Data).
